# Membrane Localization of Piezo1 in the Context of Its Role in the Regulation of Red Blood Cell Volume

**DOI:** 10.3389/fphys.2022.879038

**Published:** 2022-05-20

**Authors:** Bojan Božič, Saša Svetina

**Affiliations:** ^1^ Institute of Biophysics, Faculty of Medicine, University of Ljubljana, Ljubljana, Slovenia; ^2^ Jožef Stefan Institute, Ljubljana, Slovenia

**Keywords:** lateral distribution, membrane curvature, channel, intrinsic curvature, discocyte, membrane permeability, spontaneous curvature

## Abstract

Piezo1 is a membrane nonspecific cation channel involved in red blood cells (RBCs) in the regulation of their volume. Recently, it was shown that it is distributed on the RBC membrane in a nonuniform manner. Here it is shown that it is possible to interpret the lateral distribution of Piezo1 molecules on RBC membrane by the curvature dependent Piezo1—bilayer interaction which is the consequence of the mismatch between the intrinsic principal curvatures of the Piezo1 trimer and the principal curvatures of the membrane at Piezo1′s location but without its presence. This result supports the previously proposed model for the role of Piezo1 in the regulation of RBC volume.

## Introduction

Piezo1 is a mechanosensitive nonspecific cation membrane channel present in a variety of tissues where it plays a significant role in various physiological processes (Murthy et al., 2017; Lai et al., 2021). In the red blood cell (RBC) it is involved in regulating cell´s volume, as evidenced by the fact that mutations resulting in its gain of function cause hereditary xerocytosis, a disease characterized by the subnormal RBC volume (Zarychanski et al., 2012). RBC volume depends on cell´s content of potassium ions (K^+^) established by the balance between their Na^+^-K^+^-ATPase driven influx and their passive efflux (Hoffmann et al., 2009). It has been demonstrated that Piezo1 affects the latter, in a manner such that its transient opening causes an influx of calcium ions that subsequently activate the specific K^+^ permeating Gárdos channels (Cahalan et al., 2015). Recently, it was revealed, by high-resolution atomic force and confocal microscopy studies, that the area density of Piezo1 molecules varies along the RBC discocyte membrane in a nonuniform manner, in that it is larger than the average in regions of its poles (in dimples) and smaller than the average in the region of its rim (Dumitru et al., 2021).

A similar nonuniform lateral distribution of Piezo1 as revealed by Dumitru et al. (2021) was a crucial element of the model of a possible mechanism of the Piezo1—governed regulation of the RBC volume (Svetina et al., 2019; Svetina, 2020). The latter model was based on previously established effect of Piezo1 on the RBC efflux of potassium ions and the assumption that the Piezo1—born permeability for cations depends, on average, on the curvature of the membrane at Piezo1′s location but without its presence. The reason for such dependence was sought in the mismatch between the membrane principal curvatures and the intrinsic principal curvatures of the Piezo1 trimer. According to this type of mismatch, the Piezo1 molecules would i), because of their curved structure (Ge et al., 2012; Guo and MacKinnon, 2017; Saotome et al., 2018; Zhao et al., 2018), tend to accumulate in the RBC discocyte shape in the region of its poles, and ii), have a smaller probability to transform into their permeable state. The model predicted the establishment of a negative feedback loop between the RBC reduced volume (volume divided by the volume of a sphere with its surface area equal to the area of RBC membrane) and the average permeability of RBC membrane for the potassium ions. In this model, the action of Piezo1 on K^+^ efflux depends on the RBC discoid shape.

The aim of the present work is to demonstrate the consistency between the basic assumptions of the noted model (Svetina et al., 2019) and the observed Piezo1 membrane localization (Dumitru et al., 2021). In addition, the magnitude of the parameters that describe the interaction between Piezo1 and its bilayer environment will be estimated. The coupling between RBC shape and the lateral distribution of Piezo1 molecules will be analyzed by minimizing the membrane energy involving the bending energy of the membrane and the free energy of the freely moving membrane inclusions by a procedure developed earlier (Božič et al., 2006). In the free energy of inclusions the Piezo1—bilayer interaction will be described by the general phenomenological inclusion—bilayer interaction term (Kralj-Iglič et al., 1999) adapted for this specific case.

The section Theory and Methods will be devoted to a description of the applied theory. In Results we will first show the dependence of the system´s behavior on model parameters. The consistency of the model of Piezo1 regulated RBC volume (Svetina et al., 2019) will be checked by investigating the dependence of RBC shape on the reduced volume by using the parameters giving rise to the Piezo1 lateral distribution that agrees with the experimentally determined Piezo1 membrane location (Dumitru et al., 2021). It will be shown how the discocyte RBC shape causes Piezo1 molecules to distribute along membrane in a nonuniform manner and, also, that there is a consequent modification of the cell shape. Finally, we will comment on how these results relate to the role of Piezo1 in the regulation of RBC volume.

## Theory and Methods

In the present analysis we follow the approach developed for studying shape behavior of vesicles whose membranes contain inclusions that are free to move in the plane of the membrane (Božič et al., 2006). Vesicle shape and the corresponding lateral distribution of inclusions are obtained by solving the shape equation that is the result of the minimization of the sum of the bending energy of the membrane (*W*_b_) and the free energy of inclusions comprising a term describing their interaction with the surrounding membrane and a term due to the entropy of mixing (*F*_N_).

Membrane bending energy is defined (Canham, 1970; Helfrich, 1973) as

$$W_{b}=2k_{c}\int H^{2}dA$$ (1)

where *k*_c_ is the bending modulus, *H* the mean membrane curvature, defined as the mean of the sum of membrane principal curvature *C*_1_ and *C*_2_

$$H=\frac{1}{2}\left(C_{1}+C_{2}\right),$$ (2)

and integration runs over the cell membrane area (*A*).

The free energy of *N* membrane inclusions can be, in the limit of an ideal gas, expressed as (Kralj-Iglič et al., 1996)

$$F_{N}=\int n\left(E_{int}+k_{B}Tln\frac{n}{\bar{n}}\;\right)dA$$ (3)

where *n* is inclusion area density, $\bar{n}$ its average value (*N*/*A*), *E*_int_ the inclusion—bilayer interaction, *k*_B_ the Boltzmann constant and *T* the temperature. The limit of ideal gas for the entropy of the mixing term is appropriate because RBC proteome analysis has shown that its membrane contains about 170 (166 ± 109) Piezo1 monomers (Gautier et al., 2018). This means that there may be, on the RBC membrane area of *A* ∼ 140 μm^2^, only a little more than 50 Piezo1 trimers that form the channels. The interaction *E*_int_ can be described by the phenomenological energy term based on the mismatch between the intrinsic curvatures of the inclusion (*C*_1,incl_ and *C*_2,incl_) and membrane curvatures at Piezo1′s location but without its presence (*C*_1_ and *C*_2_). By definition, a curvature is positive when it is convex with respect to the cell interior. The general expression for the corresponding energy term, at the limit of a rigid inclusion, has been formulated by Kralj-Iglič et al. (1999) as

$$E_{\mathrm{int}}=\frac{\kappa }{2}{\left(H-H_{\mathrm{incl}}\right)}^{2}+\frac{\kappa^{*}}{2}\left[\Delta H^{2}-2\Delta H\Delta H_{\mathrm{incl}}\cos\left(2\omega \right)+\Delta H_{\mathrm{incl}}^{2}\right]$$ (4)

where *H*_incl_ = (*C*_1,incl_ + *C*_2,incl_)/2 is the mean intrinsic curvature of Piezo1 and Δ*H*_incl_ = (*C*_1,incl_ − *C*_2,incl_)/2 is a measure of the difference between the two channel intrinsic principal curvatures, Δ*H* = (*C*_1_ − *C*_2_)/2 is a measure of the difference between the two membrane principal curvatures, *κ* and *κ* ^∗^ independent interaction constants, and the angle *ω* defines the mutual orientation of the coordinate systems of the intrinsic principal curvatures of the channel and the principal curvatures of the membrane.

Expression 4 (implemented in our previous analysis (Svetina et al., 2019)) is the minimal general expression for the inclusion—membrane interaction encompassing different symmetry situations with regard to intrinsic principal curvatures of the inclusion and principal curvatures of the bilayer (Svetina, 2015). In the case of Piezo1, the middle term of its second part only counts for its conformations in which Δ*H*_incl_ ≠ 0, i.e., for example, when one of its arms has a different structure. Here we restrict the analysis to the lateral distribution of Piezo1 in its axisymmetric resting state. Expression 4 thus involves only three curvature dependent terms which are proportional to *H* ^2^, *H*_incl_ *H*, and Δ*H* ^2^. It can be further simplified because the intrinsic curvatures of the Piezo1 trimer are about three orders of magnitude larger than the principal curvatures of the membrane of the RBC discocyte. Namely, the mean curvature of the Piezo1 molecule is 100 μm^−1^ (Guo and MacKinnon, 2017) and that of the discocyte shape predicted by the minimization of membrane bending energy for the reduced volume 0.6 and membrane area 140 μm^2^ is in the range of —0.34 µm^−1^ to 0.62 µm^−1^ (Svetina and Žekš, 1989). Therefore, assuming the same order of magnitude of the interaction constants *κ* and *κ* ^*^, the terms with *H* ^2^ and Δ*H* ^2^ can be neglected. The leading term of Eqn. 4 is thus —*κH*_incl_ *H*, and we can, for the interaction term, simply take

$$E_{\mathrm{int}}=-bH,$$ (5)

its only parameter being *b* = *κH*_incl_.

It is convenient to minimize the described energy functional (*W*_tot_ = *W*_b_ + *F*_N_) in its reduced form in which all energy terms are divided by the bending energy of a spherical vesicle (8π*k*_c_) (denoted by the corresponding small letters), and all distances reduced with respect to the radius of the sphere with the membrane area *A*, i.e., *R*_s_ = (*A*/4π)^1/2^. Then

$$w_{tot}=w_{b}+p\int \frac{n}{\bar{n}}\left(-\beta h+\ln\frac{n}{\bar{n}}\right)da$$ (6)

Where *h* = *R*_s_ *H*, d*a* = d*A*/4π*R*_s_^2^,

$$p=\frac{Nk_{\text{B}}T}{8\pi k_{c}}$$ (7)

and

$$\beta =\frac{b}{k_{\text{B}}TR_{s}}$$ (8)

**FIGURE 1:**
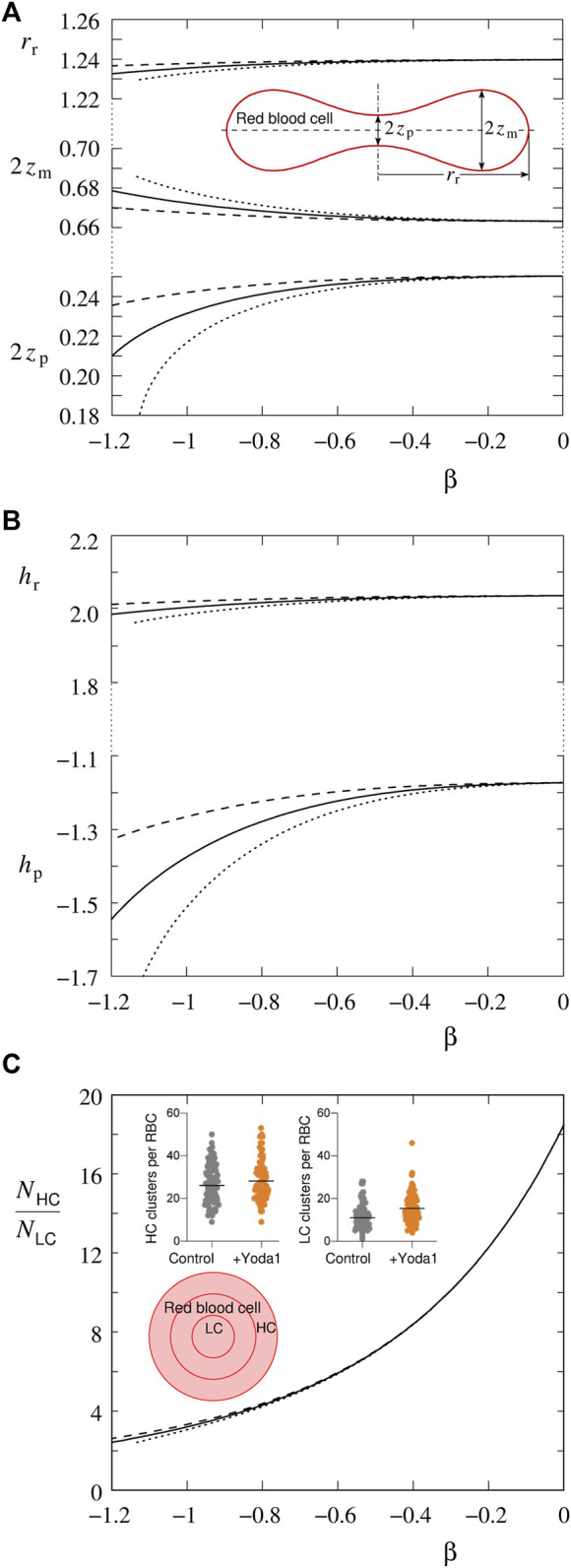
The effect of the parameter *β* on RBC shape characteristics and the ratio *N*_HC_/*N*_LC_ (for definitions, see the text and the inserts in Figures 1A,C, respectively), at different numbers of Piezo1 molecules represented by the parameter *p* (dashed line for *p* = 0.04, full line for *p* = 0.08, dotted line for *p* = 0.12). RBC reduced volume is *v* = 0.6. The distances and curvatures are given relative to *R*_s_ = (*A*/4π)^1/2^. **(A)** The dependences of the reduced distance between the poles (2*z*_p_), the largest reduced distance between the membrane above and below the discocyte symmetry plane (2*z*_m_) and the reduced radius of the rim (*r*_r_) on the parameter *β*. The insert shows the contour of the RBC axial cross-section obtained by minimization of Eqn. 1 at *v* = 0.6. The essential geometrical shape characteristics are indicated. **(B)** The reduced mean curvature at the pole (*h*_p_) and at the rim (*h*_r_). **(C)** The ratio between numbers of Piezo1 molecules in the rim region and in the pole regions (*N*_HC_/*N*_LC_). As the insert are presented slightly adapted Figures 3A,E,F of Dumitru et al. (2021). It is shown how the 2D horizontal projection of the RBC surface is divided by three concentric circles with radii *r*_r_/3, 2*r*_r_/3 and *r*_r_ that define the inner area (LC) (dimple regions), median area, and outer area (HC) (rim region). The other two pictures show, in the diction of the corresponding caption, “the abundance of Piezo1 clusters in HC and LC areas for control and Yoda1-treated RBCs”.

**FIGURE 2:**
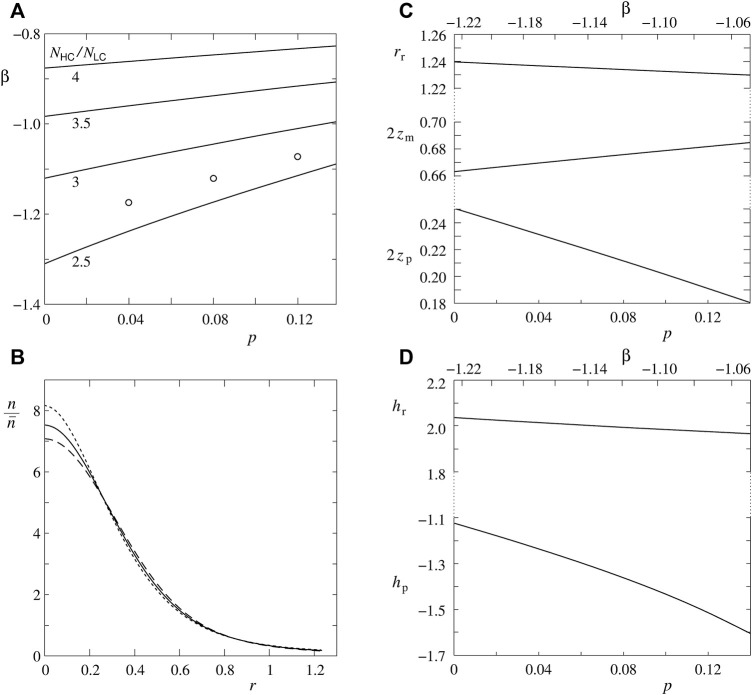
Model predictions for the effect of Piezo1—bilayer interaction on RBC shape and Piezo1 lateral distribution. **(A)** Combinations of model parameters *p* and *β* that give the indicated values of the ratio *N*_HC_/*N*_LC_. The three points indicate values of parameters *p* and *β* employed in further analyses. **(B)** Relative area density of Piezo1 molecules (*n*/ $\bar{n}$ where $\bar{n}$ is the average area density) in dependence on the reduced distance from the shape axis (*r*) at reduced volume 0.6 and at *N*_HC_/*N*_LC_ = 2.7 for three combinations of the parameter *p* (0.04, 0.08 and 0.12) and interaction constant (*β* = –1.17, –1.12 and –1.07), respectively. Type of lines is the same as for the same values of the parameter *p* in Figure 1. **(C)** RBC discocyte characteristic parameters (2*z*_p_, 2*z*_m_ and *r*_r_) obtained at reduced volume 0.6 and the ratio *N*_HC_/*N*_LC_ = 2.7 in dependence on the number of RBC Piezo1 trimers represented by the parameter *p*. **(D)** The corresponding dependence of the RBC mean curvature at the poles (*h*_p_) and at the rim (*h*_r_). In the latter two graphs the line on their top gives the corresponding scale for the parameter *β*.

The derivation of the corresponding shape equation and how it is solved for axisymmetrical shapes was described in Božič et al. (2006). The solution of the shape equation (at given values of the RBC reduced volume and the parameters *p* and *β*) comprises the axial contour of the shape (see the insert in Figure 1A) and the dependence of the relative inclusion density $\left(n/\bar{n}\right)$ as the function of the distance from the axis, as it is for instance presented in Figure 2B.

## Results

Analysis has been performed in three consecutive steps, first by looking at how an RBC discocyte shape and the corresponding lateral distribution of Piezo1 channels depend, at a given reduced volume (*v* = 3*V*/4π*R*_s_^3^, where *V* is the volume of RBC), on their number and on the strength of their interaction with the surrounding bilayer. They are represented in Eqn. 6 by the parameters *p* and *β*, respectively. This has been followed by a search of combinations of these two parameters that could give rise to the fit of the experimental results of Dumitru et al. (2021). Finally, we have observed how the system´s behavior, with the thus obtained parameters, depends on the RBC reduced volume. In this study it is assumed that Piezo1 molecules are in their low energy state as revealed by cryoEM structural analysis (Guo and MacKinnon, 2017; Saotome et al., 2018; Zhao et al., 2018).

The physiologically relevant representative RBC reduced volume is *v* = 0.6. RBC discocyte shape at and around this reduced volume is well characterized by the distance between poles (2*z*_p_), the maximal distance between the membrane above and below of the discocyte mirror plane (2*z*_m_), the distance from the rotational axis to the rim (*r*_r_), and the mean curvatures at poles (*h*_p_) and the rim (*h*_r_) (see the insert in Figure 1A showing the axial cross-section of the RBC discocyte). In Figures 1A,B it is demonstrated how these shape characteristics depend on the interaction between Piezo1 and the surrounding bilayer. The increase of its strength (the absolute value of the parameter *β*) causes the pole-to-pole distance to be shorter. The radius of the rim also diminishes but to a much less relative extent. Correspondingly, in the investigated interval of the parameter *β*, the membrane mean curvature at the poles becomes more negative, and the mean curvature at the rim does not change appreciably (Figure 1B). All these dependences are more pronounced at larger number of Piezo1 channels (*N*), represented by the parameter of the model *p*, and thus also at larger value of their average areal number density ( $\bar{\;n}$ ). The chosen three values of the parameter *p* are supposed to lie within a realistic range of RBC membrane bending stiffness, *k*_c_, and a realistic range of the number of Piezo1 molecules, *N*. For example, by taking *k*_c_ = 2.0 10^–19^ J (Hwang and Waugh, 1997) and *p* = 0.08, we get at *T* = 300 K (see Eqn. 7) the number of Piezo1 trimers *N* = 100 which is within the range obtained by the proteome analysis (Gautier et al., 2018). Piezo1 lateral inhomogeneity is manifested, as evidenced by Dumitru et al. (2021), by the ratio between the number of Piezo1 molecules in the rim region (*N*_HC_) and dimple regions (*N*_LC_) (see the insert in Figure 1C). The numbers *N*_HC_ and *N*_LC_ are obtained by the integrals of the areal number density (*n*) over the corresponding areas ( ∫ndA ). The ratio *N*_HC_/*N*_LC_ is, as a function of the absolute value of the parameter *β*, decreasing (Figure 1C). Here it has to be noted that, at zero interaction constant where the distribution is uniform, the plotted value 18.5 reflects the ratio between the respective membrane areas (*A*_HC_/*A*_LC_). Interestingly, the ratio *N*_HC_/*N*_LC_ is, except for rather large absolute values of the parameter *β*, practically independent on the number of molecules.

The next set of figures (Figure 2) is devoted to the estimate of model parameters *β* and *p* for which the ratio between the number of Piezo1 molecules in the rim and pole regions *N*_HC_/*N*_LC_ agrees with the measured value of Dumitru et al. (2021). In Figure 2A are shown combinations of model parameters *β* and *p* for which are obtained the same ratios *N*_HC_/*N*_LC_. In accord with the dependence of *N*_HC_/*N*_LC_ on *β* (Figure 1C), the effect of the number of Piezo1 molecules is not very significant at smaller absolute values of the parameter *β*, however, the situation becomes different below its value of about —0.8. At larger number of Piezo1 molecules the same ratio *N*_HC_/*N*_LC_ is obtained at much smaller absolute values of the parameter *β*. The results of Dumitru et al. (2021) show the numbers *N*_HC_ and *N*_LC_ (by assuming that these numbers are proportional to the number of detected Piezo1 clusters) to be in different cells of the RBC population considerably different (see the insert in Figure 1C). However, it is possible to use for the measure of the ratio *N*_HC_/*N*_LC_ its value obtained for a hypothetical “average” cell that has the numbers *N*_HC_ and *N*_LC_ proportional to the corresponding experimentally determined average values of the numbers of Piezo1 clusters. These values are for the controls (grey points in the insert of Figure 1C presented pictures) 27 for HC and 10 for LC, so that *N*_HC_/*N*_LC_ = 2.7. By accepting this value and taking *p* = 0.08, the parameter *β* is estimated to be —1.12. In Figure 2A the location of this point is shown by a circle. The other two points in Figure 2A indicate the values of the parameter *β* for *N*_HC_/*N*_LC_ = 2.7 at *p* = 0.04 and *p* = 1.2. It is of interest to see how different combinations of parameters *p* and *β* which predict the same ratio *N*_HC_/*N*_LC_ affect the predicted Piezo1 lateral distribution and RBC shape. This is shown in Figures 2B–D. The choice of parameters *β* and *p* affects the predicted Piezo1 areal density the most in the region of poles (Figure 2B). This is also reflected in the pole-to-pole distance (2*z*_p_) (Figure 2C) and the pole curvature (*h*_p_) (Figure 2D) which depend on the constant *N*_HC_/*N*_LC_ combination of parameters *β* and *p* much stronger than the discocyte shape characteristics pertaining to the rim region. The described dependences are additional evidence that, with respect to the number of RBC Piezo1 channels, the most sensitive part of the RBC discocyte shape is its dimple region.

**FIGURE 3:**
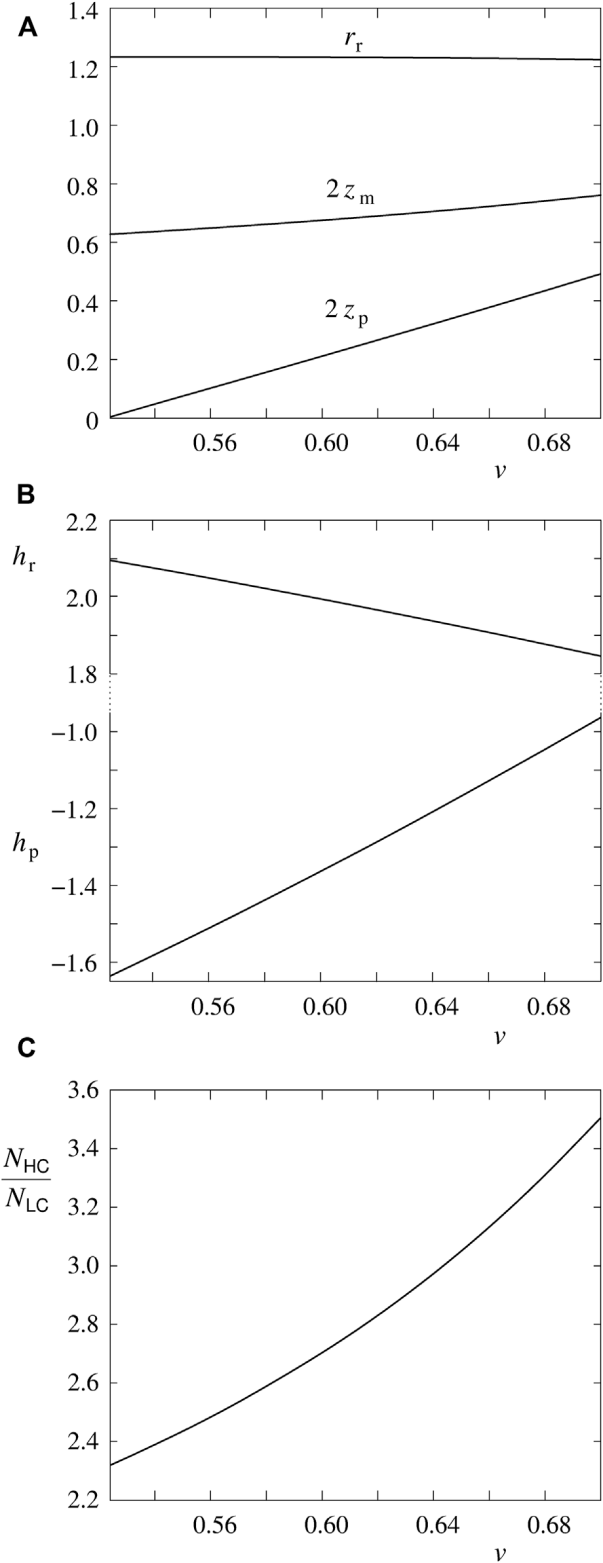
The dependence of the behavior of the treated system on the RBC reduced volume *v*. The system´s parameters presented are the same as in Figure 1, i.e., 2*z*_p_, 2*z*_m_ and *r*_r_, **(A)**, *h*_p_ and *h*_r_ **(B)**, and the ratio *N*_HC_/*N*_LC_ **(C)**. The calculations are made for the values of model parameters *β* = —1.12 and *p* = 0.08.

The last set of figures (Figure 3) shows the dependence of the behavior of the treated system on the RBC reduced volume. We choose for the parameters *β* = —1.12 and *p* = 0.08. The range of interest for the reduced volume is from *v* = 0.7 to its value where, in this case, the distance between poles becomes zero (*v* = 0.522). The results accord with expectations: pole-to-pole distance is decreasing (Figure 3A), the mean curvature at poles is becoming more negative while the one at the rim is becoming more positive (Figure 3B). The ratio between the number of Piezo1 molecules in the rim and pole regions is, in dependence on *v*, decreasing (Figure 3C).

## Discussion

We first comment on the presented graphs and then, in a more general sense, put this study in the frame of some other recent related analyses.

The results in Figure 1 show that the interaction between Piezo1 and the bilayer has an effect both on RBC shape and the lateral distribution of channels. Piezo1 molecules thus act as shape sensing and shape forming entities. Such a mutual effect on each other of vesicle shape and lateral distribution of membrane inclusions or membrane binding molecules has been studied a lot for shapes composed of sections with constant curvatures. An example is a cylindrical tether pulled out of a vesicle aspirated into a pipette, where it is possible to describe shape by a simple geometrical model (Simunovic et al., 2016). RBC discocyte exhibits a continuously varying mean curvature from its negative value at poles to its positive value at the rim. The results in Figure 1 show why it is, in such cases, crucial to apply the theoretical approach developed for general axisymmetrical shapes of vesicles with inclusions (Božič et al., 2006). The effect of Piezo1 on the shape is, in the region of the rim, much less pronounced than in the dimple region where there is also a much larger area density of Piezo1 molecules.

The results presented in Figure 2 imply that it is possible to interpret the distribution of Piezo1 molecules observed by Dumitru et al. (2021) over the RBC membrane solely on the basis of the curvature dependent interaction between Piezo1 molecules and the surrounding bilayer. The system´s behavior depends on the parameters *β* and *p* which represent the strength of the Piezo1—bilayer interaction, and the effects of the contribution of its entropy, respectively. The estimated value of the ratio between the number of Piezo1 molecules in the rim region and dimple regions (*N*_HC_/*N*_LC_) is in the range of the values of parameters *β* and *p* where its value (2.7) can be obtained by a wide range of their combinations (Figure 2A). A better estimate of the values of parameters *β* and *p* could be obtained by measuring Piezo1 areal density with a better spatial resolution. From the comparison of the three predicted areal density distributions of Piezo1 molecules (Figure 2B) it can be concluded that the densities mostly differ within the regions of poles. A better spatial resolution should therefore be applied primarily within these regions.

The Piezo1—bilayer interaction was, in this work, described in terms of a phenomenological interaction term. It will be informative to relate this term to different basic contributions to the energy of the system. Such interactions have been studied theoretically, for example by determining due to the inclusion increased bilayer bending energy (Phillips et al., 2009). Up to now the Piezo1—bilayer interaction term has been, in this sense, calculated for a Piezo1 molecule embedded into a flat tensed membrane (Haselwandter and MacKinnon, 2018). It was shown how, due to the curved structure of Piezo1, a membrane footprint is formed that extends far beyond the size of a molecule. The extension of this theory to cases where Piezo1 is embedded in an already curved membrane could give an estimate of the value of the parameter *β*.

The results presented in Figure 3 are relevant with regard to the recently proposed model of the role of Piezo1 in the regulation of RBC volume (Svetina et al., 2019; Svetina, 2020). The crucial element of this model was the nonuniform distribution of Piezo1 molecules due to their curvature dependent interaction with the surrounding bilayer. Due to lack of the appropriate data, the procedure was simplified by the assumption that Piezo1 molecules sense the curvature at the discocyte poles. Figure 3C is an indication that the system behaves, in qualitative terms, in the same manner. In consequence, instead of relying on an assumption, it will be possible, in further development of this modeling, to apply the Piezo1 area distribution obtained experimentally (Dumitru et al., 2021). Another improvement of the model would be the predicted effect of the Piezo1 lateral distribution on the pole-to-pole distance (Figure 1A). In this respect it is important to note that, in the treated case, the distribution and discocyte shape act on each other in a manner of a positive feedback. The more Piezo1 molecules reside in the region of poles, the larger is the absolute value of the pole curvature; the larger is the latter, the more Piezo1 molecules tend to reside in this region.

The occurrence of the nonuniform distribution of Piezo1 molecules implies strongly that they are laterally mobile. This appears to be in conflict with the notion of the effect of a spectrin network on their mobility (Dumitru et al., 2021). However, it can be assumed that Piezo1 molecules are, because of the formed footprint (Haselwandter and MacKinnon, 2018), corralled by the spectrin network which can cause their effective diffusion constant on the RBC membrane to be considerably smaller than in membranes without such a skeleton.

This analysis is a strong indication that it is the curvature dependent Piezo1–bilayer interaction that is responsible for the observed Piezo1 lateral distribution on the RBC membrane. Nevertheless, one has to be aware of its possible limitations. For example, the described simple system seems to be inadequate for the interpretation of the effect Yoda1 on the discocyte lateral distribution of Piezo1 (Dumitru et al., 2021). In the presence of Yoda1 the ratio *N*_HC_/*N*_LC_ extracted from the mean values of counted Piezo1 clusters (insert in Figure 1C) is smaller (1.9) than in the control (2.7) which could be understood as an indication that Yoda1 causes Piezo1 to be curved more than in its absence, contrary to the common view that it causes Piezo1 structure to transform in the direction of its less curved open state. This discrepancy could be the result of our assumption that all Piezo1 molecules reside in their state with the lowest energy, as revealed by cryoEM (Guo and MacKinnon, 2017; Saotome et al., 2018; Zhao et al., 2018). But it could also happen that a Piezo1 molecule embedded in a curved membrane at a given curvature switches into one of its other stable conformations with smaller intrinsic curvatures. A hypothesis can thus be made that Yoda1 causes a shift of such a critical membrane curvature. However, at present there is not enough data on structures of other than the resting state of Piezo1 to confirm this interpretation. Furthermore, it is also necessary to take into consideration that there are several other factors that have an effect on the RBC discocyte shape. One is the difference between the areas of the two bilayer leaflets. It is known, from theoretical analyses, that the pole-to-pole distance increases as a function of such an area difference (Svetina and Žekš, 1989). Recently it has been revealed that the pole-to-pole distance also depends on the activity of the RBC´s actin-myosin system (Smith et al., 2018). In these respects the part of the present analysis related to the establishment of the RBC discocyte shape is far from complete.

In relation to the present work, the effects of the actin-myosin system deserve special attention. It has been shown that actins and myosins are denser in the region of the RBC dimple (Alimohamadi et al., 2020). The present study suggests that this property could be the consequence of the accumulation of Piezo1 molecules in this region. The formation of membrane footprints (Haselwandter and MacKinnon, 2018) indicates that the effective lateral compressibility of the membrane is larger than in their absence. Consequently, it varies along the RBC membrane in correspondence to the Piezo1 lateral distribution. Under condition of the lateral compression of the membrane involving Piezo1 footprints, it can thus be expected that it causes the increase of their extra membrane area, implying that membrane lateral compressibility has a component proportional to the Piezo1 area density.

The mismatch principle can be considered as one of the ways to interpret consequences of the interaction between membrane bilayer and shape sensing and forming membrane proteins (reviewed in Tsai et al., 2021). In this study it has been realized that, in the case of RBC Piezo1, because of the intrinsic mean curvature of Piezo1 being orders of magnitude larger than that of the RBC membrane, its product with the interaction constant (parameter *b*) matters more than this constant (*H*_incl_) by itself. The interaction term thus involves only a single constant, which can be considered as a macroscopic material constant. The sum of the bending energy (Eqn. 1) and contributions of the interaction term (Eqn. 5) can be transformed in this case into an expression for the area density of the bending energy of a membrane $2k_{c}{\left(H-C_{0}^{eff}/2\right)}^{2}\;$ , by *C*_0_^eff^ = *nb*/*k*_c_ considered as a laterally variable effective spontaneous curvature. “Effective” because it is the result of the strength of the interaction and is not related to a certain real radius of a sphere. It has also to be pointed out that the calculations presented here belong to the “mismatch” type and not to the “spontaneous curvature” type modeling (Tsai et al., 2021), because the energy functional to be minimized (Eqn. 6) still involves the entropy part of the free energy of inclusions. However, the concept of spontaneous curvature modeling could be applied if the location of inclusions were fixed, for example if shape transformations would occur in times much shorter than is the characteristic time for the system´s equilibration, due to Piezo1 lateral diffusion.

## Data Availability

The raw data supporting the conclusions of this article will be made available by the authors, without undue reservation.
